# U-SPECT-BioFluo: an integrated radionuclide, bioluminescence, and fluorescence imaging platform

**DOI:** 10.1186/s13550-014-0056-0

**Published:** 2014-10-11

**Authors:** Matthias N van Oosterom, Rob Kreuger, Tessa Buckle, Wendy A Mahn, Anton Bunschoten, Lee Josephson, Fijs WB van Leeuwen, Freek J Beekman

**Affiliations:** 1 Radiation, Detection and Medical Imaging, Delft University of Technology, Mekelweg 15, Delft, 2629, JB, the Netherlands; 2 Interventional Molecular Imaging Laboratory, Department of Radiology, Leiden University Medical Center, Leiden, the Netherlands; 3 Centre for Translational Nuclear Medicine and Molecular Imaging, Massachusetts General Hospital, Harvard Medical School, Boston, USA; 4 MILABS, Utrecht, the Netherlands; 5 Department of Translational Neuroscience, Brain Center Rudolf Magnus, University Medical Center Utrecht, Utrecht, the Netherlands

**Keywords:** Bioluminescence imaging, Fluorescence imaging, Multimodal molecular imaging, SPECT, Small animal, Nuclear medicine

## Abstract

**Background:**

*In vivo* bioluminescence, fluorescence, and single-photon emission computed tomography (SPECT) imaging provide complementary information about biological processes. However, to date these signatures are evaluated separately on individual preclinical systems. In this paper, we introduce a fully integrated bioluminescence-fluorescence-SPECT platform. Next to an optimization in logistics and image fusion, this integration can help improve understanding of the optical imaging (OI) results.

**Methods:**

An OI module was developed for a preclinical SPECT system (U-SPECT, MILabs, Utrecht, the Netherlands). The applicability of the module for bioluminescence and fluorescence imaging was evaluated in both a phantom and in an *in vivo* setting using mice implanted with a 4 T1-luc + tumor. A combination of a fluorescent dye and radioactive moiety was used to directly relate the optical images of the module to the SPECT findings. Bioluminescence imaging (BLI) was compared to the localization of the fluorescence signal in the tumors.

**Results:**

Both the phantom and *in vivo* mouse studies showed that superficial fluorescence signals could be imaged accurately. The SPECT and bioluminescence images could be used to place the fluorescence findings in perspective, e.g. by showing tracer accumulation in non-target organs such as the liver and kidneys (SPECT) and giving a semi-quantitative read-out for tumor spread (bioluminescence).

**Conclusions:**

We developed a fully integrated multimodal platform that provides complementary registered imaging of bioluminescent, fluorescent, and SPECT signatures in a single scanning session with a single dose of anesthesia. In our view, integration of these modalities helps to improve data interpretation of optical findings in relation to radionuclide images.

## 1 Background

Studies of human disease in animal models often provide an essential link between chemistry, fundamental research at the molecular level, and the development/evaluation of new diagnostic and therapeutic methods. Molecular imaging modalities such as single-photon emission computed tomography (SPECT), positron emission tomography (PET), bioluminescence, and fluorescence provide a means to generate a view into the *in vivo* situation [1],[2].

SPECT and optical technologies are complementary in terms of resolution, speed, quantitative accuracy, and tracer availability [3]-[5]. Independent of the source depth, modern small animal SPECT systems can be used for longitudinal and quantitative imaging studies of dynamic processes in small structures (resolution <0.25 mm) [6]. Alternatively, optical imaging provides a rapid and low-cost non-radioactive imaging and enables longitudinal studies of superficial lesions. The resolution of fluorescence imaging also enables the (*ex vivo*) microscopic visualization of molecular/cellular processes [7]. Potentially, a vast amount of optical tracers from molecular cell biology can be translated to *in vivo* use. Alternative to a use in combination with dedicated tracers, optical imaging can also be used to detect transfected (tumor) cell lines that contain e.g. luciferase or one of the fluorescent proteins [8], enabling the (longitudinal) monitoring of disease spread and progression in animal models. The main disadvantages for optical techniques are the significant scatter and absorption of photons by tissue/structures in the animal body. Fluorescence techniques additionally suffer from tissue autofluorescence, resulting in an unwanted background signal. These negative features can be partly overcome by using emissions in the near-infrared (NIR) spectrum, but unfortunately optical imaging cannot equal the detailed view and quantitative accuracy that SPECT provides at larger depths. One of the great challenges today is to place these two modalities in perspective and to determine the added value of optical imaging within the field of nuclear medicine [9].

When multimodal tracers with both a radioactive and fluorescent signature are used, a single tracer can provide ‘best of both worlds’. Such tracers are reported with a peptide, monoclonal antibody, or nanoparticle basis [10]-[12]. Combined radioactive and fluorescence imaging has already provided added value in clinical surgical guidance studies [13],[14].

Next to the development of multimodal tracers that combine two signatures, SPECT and optical modalities can also be combined on a single device with a single user interface. In this way, logistics, including anesthesia, are simplified and may improve animal welfare. Because animal positioning is no longer an issue with respect to co-registration of the imaging results, the complementary value of the different modalities can be exploited to its full extent; e.g. bioluminescent tumor cell localization combined with tracer distribution imaging (fluorescence and/or nuclear).

While other groups mainly focus on integrating nuclear imaging with three-dimensional (3D) fluorescence optical tomography (FOT) imaging [15]-[18] or fluorescence-mediated tomography (FMT) [19], we consider optical imaging, both bioluminescence and fluorescence, as intrinsically superficial technology, currently most suitable for planar imaging. Today, planar imaging is applied in the far majority of optical imaging studies in preclinical research since optical tomography is still highly challenging due to the ill-posed nature of optical data caused by strong light absorption and diffusion [20]. Therefore, we pursued integration of planar optical imaging with SPECT. We developed and tested a low-cost prototype system consisting of an add-on planar bioluminescence/fluorescence optical module for a dedicated small animal SPECT device (U-SPECT-II, MILabs B.V., Utrecht, the Netherlands) [21]-[24].

## 2 Methods

### 2.1 Optical module for small animal SPECT

**Figure 1:**
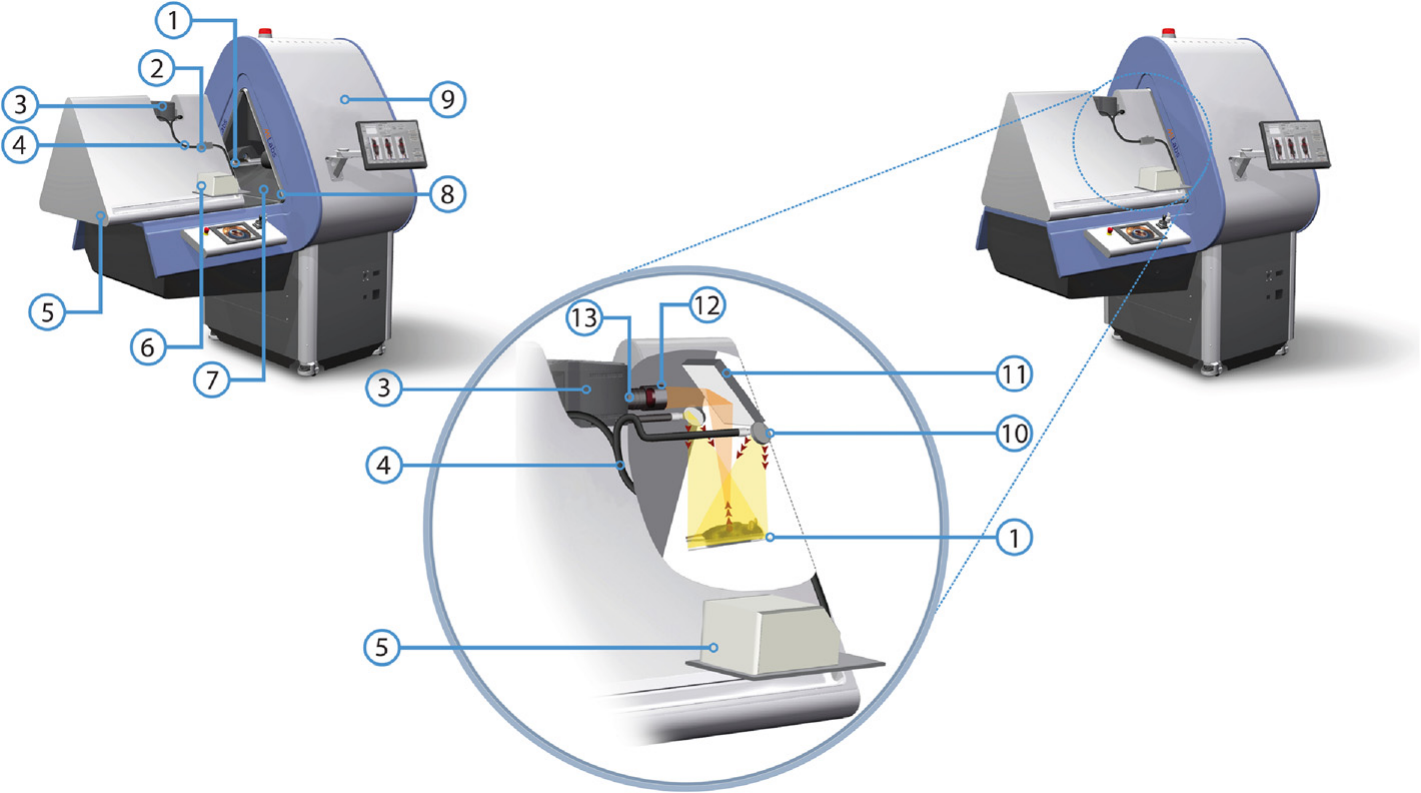
**Overview of the U-SPECT-BioFluo platform. (1)** Animal bed. **(2)** Excitation filter box. **(3)** Camera. **(4)** Optical fiber bundle. **(5)** Dark box. **(6)** Light source. **(7)** Black ABS-plastic insert. **(8)** U-profile light lock. **(9)** U-SPECT. **(10)** Mirrors for excitation light. **(11)** Mirror directing light from the bed to the camera. **(12)** Lens hood and emission filter holder. **(13)** Camera lens.

The prototype optical imaging (OI) module was fitted onto the U-SPECT-II [21],[22] installed at the LUMC (Leiden, the Netherlands) as is shown in Figure 1. The optical module consists of three main components: 1) a light tight ‘dark box’ ⑤, 2) a very sensitive CCD camera ③, and 3) a bright light source ⑥. Details about these components will be given in later paragraphs. The dark box was designed in such a way that when the module is in ‘open’ position, the handling of the animals in the bed of the U-SPECT is not hampered. When the module is ‘closed’, the CCD camera on top of the box is shielded from ambient light and can produce a total-body top-view bioluminescent image of the animal via a mirror ⑪. For photographic and fluorescence imaging, the animal is illuminated by the light source via two optic fibers ④ entering the box and small mirrors ⑩ reflecting the light onto the bed ①. Excitation and emission light filters, well adapted to the spectral profile of the fluorescent dye under study, can be added to the system.

For SPECT scans, the animal bed is moved by an XYZ stage into the U-SPECT ⑨ collimator. For the OI position, the bed is held in the same position as used for placing an animal in the bed (U-SPECT ‘bed eject position’). Alternatively, by mounting the OI module on a separate ‘docking station’, it can also be used as an independent optical imaging device. The docking station allows for parallel use of the SPECT and OI module.

#### 2.1.1 Dark box

The dark box uses a rail system to slide over the table, enabling animal placement. The sliding mechanism consists of a U-profile mounted on the box and rails ⑧ mounted on the table that contains the XYZ stage to move the bed mounted at the front side of the U-SPECT. When the box slides to its closed position, it is lowered by about 1 cm. In this way, the U profiles lock over the rails. Blackboard paint has been applied on the rails and the U-profile to ensure the light tightness in locked position.

To further block ambient light, all joints on the box have been sealed with Sikaflex-221 (Sika AG, Baar, Zug, Switzerland) sealant. Blackboard paint is applied to the inside of the box to avoid light reflections. A black ABS-plastic insert ⑦ has been fitted to cover the entrance of the U-SPECT to prevent leakage of ambient light into the dark box and to avoid reflections from the metallic SPECT entrance. This insert was made with a tight fit to the entrance of the existing scanner, leaving maximum space to handle the animals on the bed. With the insert in place, the U-SPECT vision cameras can still be used to select the region of interest (ROI) in the U-SPECT acquisition software [21],[22]. Remaining light leaks measured inside the closed black box were closed using tape sealants.

#### 2.1.2 Camera, lens, and mirror

To allow for the detection of weak bioluminescence and fluorescence signals, an Andor iKon-M 912BV scientific CCD camera (Andor Technology plc., Belfast, UK) with a very low dark current level was used for recording OI images. This camera is based on a CCD77-00 (e2v Technologies Ltd., Essex, UK) with 512 × 512 pixels (24 × 24 μm) and has a quantum efficiency between ~50% and ~95% in the wavelength region of 400-900 nm. With the 16-bit camera read-out at a rate of 50 kHz, the read noise was below 2 counts/pixel. By means of an air-cooled Peltier element, the CCD operating temperature was set to −65°C, reducing the dark current to ~0.006 elec/pix/s. The camera was fully controlled by the accompanying Andor Solis software.

The CCD camera was equipped with a Fujinon (Fujifilm Corp., Tokyo, Japan) CF25HA-1 lens ⑬. This lens has a fixed focal length (F) of 25 mm. To collect as much of the emitted luminescence as possible, we selected a lens whose diaphragm could be set as large as F/1.4. An emission filter holder ⑫ (50 mm diameter) was mounted in front of the lens. A lens hood was integrated with the filter holder to avoid stray light entering the lens/filter combination.

A mirror was placed above the animal bed (Figure 1) to direct the emitted light to the CCD camera and generate a top-view image of an entire mouse. The selected Edmund Optics enhanced aluminum mirror (Edmund Optics Inc., Barrington, NJ, USA) has a high reflective index in the wavelength region of interest: between ~85% and ~98% for wavelengths larger than 450 nm.

#### 2.1.3 Fluorescence excitation light

To excite fluorescent dyes, an illumination setup was constructed to expose the whole animal (bed in OI position) with a relatively high intensity of (excitation) light at the appropriate wavelength.

An MI-150 fiber optic illuminator (Edmund Optics Inc., Barrington, NJ, USA) with a 150-W EKE halogen light bulb produced a high intensity bundle of light (450 to 800 nm), covering our wavelength range of interest for fluorescence imaging. Light from the illuminator was guided to an excitation filter box ② using a 0.25 inch glass fiber bundle guide. In the filter box, the spectrum of the excitation light can be tailored to a particular dye using a 12.5-mm diameter filter. After the filter, the light was split into two fiber bundles that enter the dark box next to the camera. The light from the fibers was reflected by two small mirrors above the bed to illuminate the mouse from two directions. Light leakage at the fiber bundle entrance points was prevented by applying cable glands.

### 2.2 Imaging with U-SPECT-BioFluo

With the U-SPECT-BioFluo device bioluminescence, fluorescence, and SPECT scans of a mouse can be made in a sequential fashion. The SPECT measurement settings are setup as described earlier for the U-SPECT [21],[22]. To perform the optical measurements, the OI module is closed and the animal bed is moved to the OI position. After positioning, grayscale photographs and bioluminescent and/or fluorescent images can be recorded by the CCD camera and stored with the Solis software for further offline analysis with Matlab (MathWorks Inc., Natick, MA, USA).

#### 2.2.1 Grayscale photograph

Grayscale images are recorded by the CCD camera to provide an anatomical context for the bioluminescence, fluorescence, and SPECT images. The grayscale images are recorded under white light illumination without the use of emission and excitation filters. To avoid saturation of the CCD image for our minimal exposure time (50 ms), the lens diaphragm is set to F/22 and the iris of the light source is set to a minimum. The F/22 diaphragm allows for sharp photographs of the animal in OI position.

#### 2.2.2 Bioluminescence imaging

The bioluminescence images are recorded without the use of excitation light and an emission filter. To provide total darkness in the module, the fiber optic illuminator is switched off and a light stop is put in the excitation filter box to block light entering the module via leaks in the illuminator. To collect a maximum of bioluminescent photons in a reasonable exposure time (<90 s), the largest possible lens diaphragm (F/1.4) is used.

#### 2.2.3 Fluorescence imaging

During fluorescence imaging, the object or animal is continuously illuminated using an appropriate excitation filter for the dye under study. With a specific emission filter in front of the CCD camera lens, only the emitted fluorescent signal is recorded by the camera.

The CCD exposure time was kept to a minimum by using the largest possible lens diaphragm (F/1.4) and fully opening the iris of the illuminator. During imaging, the exposure time was adjusted to obtain images in which the brightest fluorescence spots have pixel values larger than half the full pixel ADC range.

#### 2.2.4 Illumination calibration

The measured images are corrected for the non-uniform illumination pattern at the mouse bed by making calibration measurements. This is done by making an image (without emission filter) of the illumination pattern using a flat sheet of white paper in the OI position. The corresponding iris, diaphragm, and excitation filter are used for the grayscale or fluorescence calibration. The exposure time has to be adjusted to avoid CCD saturation. Since the bioluminescent images do not require illumination, they do not require such a calibration.

#### 2.2.5 Image processing

The optical images from the CCD camera are processed offline with Matlab. Both the fluorescence and photographic calibration images are blurred with a Gaussian filter of 7.30 mm full width at half maximum (FWHM) to suppress small scale non-uniformities of the calibration paper sheet itself. The resulting calibration images are subsequently normalized by scaling their maximum pixel value to 1. The photographic and fluorescent images are corrected for the non-uniform illumination by dividing them by the corresponding normalized calibration image.

3D SPECT images are reconstructed from the SPECT list-mode data with MILabs reconstruction software version 2.38. Proper energy and background windows are set and data is reconstructed to an isotropic voxel grid of 0.2 mm using a pixel-based ordered subset expectation maximization (POSEM) algorithm [25] which includes compensation for distance-depending blurring [26]. No attenuation correction is applied. For comparison to the optical images, a (2D) SPECT sum image is made by summing the voxel values along the vertical direction. For anatomical reference, the bioluminescence, fluorescence, and SPECT sum images are shown in color on top of the grayscale photographic image for pixel values above an adjustable threshold. For bioluminescence and fluorescence, this is straightforward as the same CCD camera is used to record these images. Overlay of the SPECT sum image was done by matching the mouse contours to the grayscale images of the mouse.

### 2.3 Performance characterization

To evaluate the multimodal imaging capabilities of the U-SPECT-BioFluo setup, pilot experiments were performed using a phantom and tumor-bearing mice injected with a multimodal tracer. Optical images were also acquired and processed on an IVIS Spectrum with Living Image software (Caliper Life Sciences Inc., Hopkinton, MA, USA). Bioluminescence imaging on the IVIS was done with open filter settings.

The fluorescent dye CyAL-5.5_b_ (λ_ex,max_ =674 nm; λ_em,max_ =693 nm) [27] was used for the phantom and the multimodal tracer. For the fluorescence measurements on the IVIS, the (built-in) 640-nm excitation and 680-nm emission filters were selected. Their bandwidths were respectively 30 nm and 20 nm. However, for the OI module we selected a commercial filter (Edmund Optics TechSpec Fluorescence Bandpass, Barrington, NJ, USA) with a center wavelength of 692 nm and wider bandwidth of ~40 nm to match the dye’s emission peak and to detect a larger part of the emission spectrum. From the same filter series another filter with ~40 nm bandwidth was used to excite this dye. To minimize the spectral overlap of the excitation and emission filters, an excitation center wavelength of 624 nm was selected. The excitation light spectrum of the filter was largely overlapping the dye’s absorption spectrum. Both commercial filters have a 93% transmission within their passbands and a blocking factor >10^6^ for wavelengths outside their passbands. This blocking factor is lower for non-perpendicular incident light. So, some excitation light reflected by the object and incident on the emission filter might still pass that filter causing the so-called excitation light leakage background in fluorescence images,

#### 2.3.1 Phantom measurements

**Figure 2:**
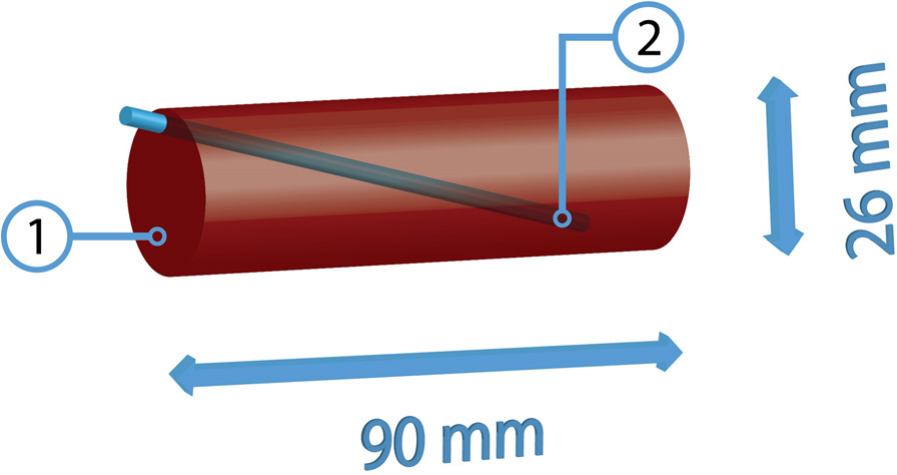
**Schematic diagram of the tissue simulating gel phantom. (1)** Phantom. **(2)** Fluorescent and radioactive capillary.

A phantom was generated, via a slightly modified procedure as described by Pleijhuis et al. [28], to evaluate the attenuation and resolution for the fluorescence and SPECT signals as function of tissue depth. This phantom (Figure 2) consisted of a cylinder of tissue simulating gel, in which a capillary, uniformly filled with a fluorescence/SPECT compound, was inserted under an oblique angle.

The gel consisted of a 1% (w/w) agarose solution that was prepared by dissolving agarose (Roche, Basel, Switzerland) in 50 mL water under continuous heating and stirring. After cooling down to 40°C, 170 μM hemoglobin from bovine blood (Sigma Aldrich, St. Louis, MO, USA) was added to the solution to obtain an optically tissue equivalent gel [28]. The solution was poured into a 50-mL tube (Falcon, BD Biosciences, San Jose, CA, USA). After further cooling and solidification, the tube was removed and the gel was given blunt ends using a scalpel.

A 200-μM CyAL-5.5_b_ solution was mixed 3:1 with a ^99m^Tc-eluate (~1.3 GBq/mL). A glass capillary (inner diameter 0.9 mm) containing 50 μL of this mixture and closed by epoxy glue [29] was inserted into the gel. Prior to inserting the capillary in the phantom, we used the blue coloration to visually confirm that the fluorescent dye (CyAL-5.5_b_) was uniformly distributed in the capillary.

The phantom was placed in the U-SPECT-BioFluo bed, and a grayscale and a fluorescence image were recorded. Subsequently, a 20-min SPECT scan was made with the ROI containing the entire phantom. At last, fluorescence measurements were made on the IVIS for control.

#### 2.3.2 In vivo measurements

Mouse scans were performed to interpret the different bioluminescent, fluorescent, and SPECT images for a practical preclinical case series of four animals. Tumor lesions were generated in Balb/c nude mice (6 to 8 weeks of age) by transplanting 4 T1-luc + tumor cells (0.25 · 10^5^), with firefly luciferase gene expression, into the fourth mammary fatpad [30].

For fluorescence and SPECT imaging, the previously reported multimodal tracer ^111^In-MSAP-RGD [30], containing the above mentioned CyAL-5.5_b_ dye, was used. Twenty-four hours prior to imaging, the mice were injected intravenously with this tracer (40 μg, 18 nmol, 10 MBq).

**Figure 3:**
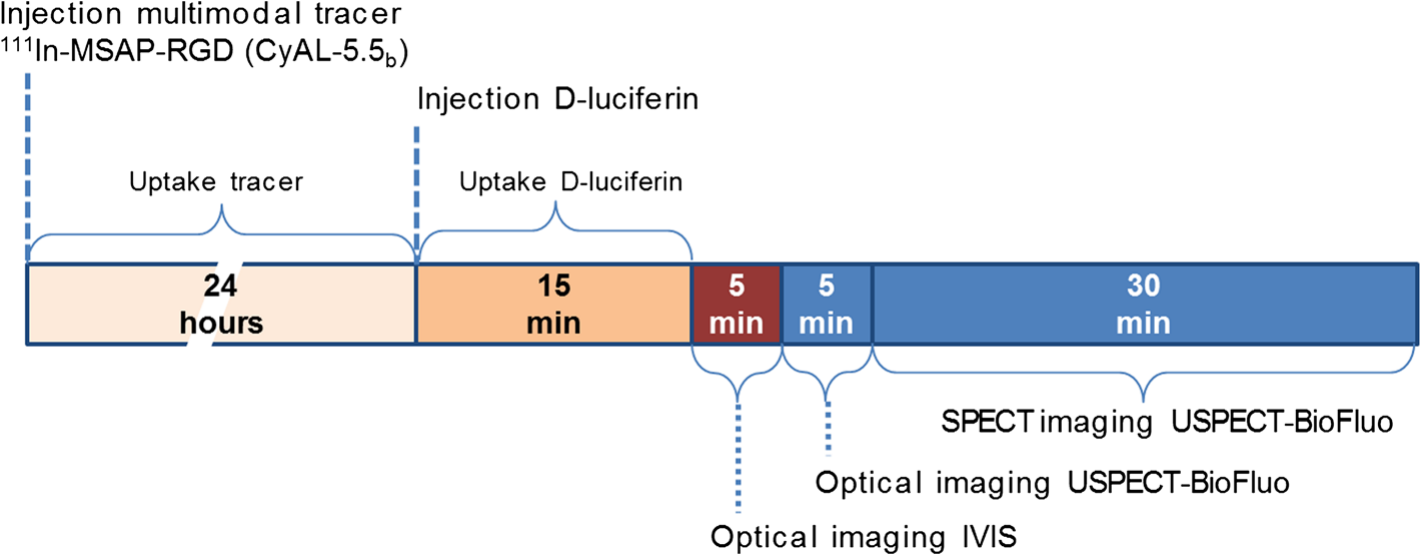
**Protocol timeline for** ***in vivo*** **measurements.** The injection moments of ^111^In-MSAP-RGD (CyAL-5.5_b_ dye), D-Luciferin, and subsequent imaging periods are indicated.

For bioluminescence imaging, the mice were injected intraperitoneally with 150 mg/kg D-Luciferin (Xenogen Corp., Alameda, CA, USA) in PBS. Ten to twenty minutes after this injection, the mice were placed in the transparent U-SPECT-BioFluo bed and first imaged on the IVIS. Afterwards, the bed with the mouse was immediately clicked on the stage of the U-SPECT-BioFluo for optical imaging followed by 30 min of SPECT imaging. The mice were anesthetized by means of Hypnorm/Dormicum/water (1:1:2; 5 μL/g i.p.). The protocol timeline is shown in Figure 3. These animal experiments were performed in accordance with Dutch welfare regulations and approved by the ethics committee of the Leiden University Medical Center under reference 2013/12189.

## 3 Results

After installing the optical module on the U-SPECT, the optical image pixel size for the top-view image of the bed was found to be 0.36 mm. We verified the light tightness of the closed dark box by recording an image with a large lens diaphragm and an exposure time of 60 s, but without any optical filters or light source. This measurement resulted in a zero mean ambient light background level (standard deviation: 1.3 counts/pixel).

### 3.1 Phantom measurements

**Figure 4:**
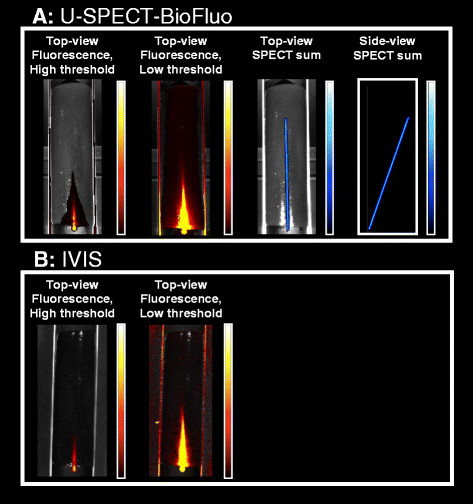
**Phantom imaging results. (A)** Fluorescence (500 ms exposure time) and SPECT imaging on the U-SPECT-BioFluo. **(B)** Fluorescence imaging on the IVIS (1s exposure time).

In Figure 4, the fluorescence and SPECT imaging results of the cylindrical phantom with the thin capillary source are shown both for the U-SPECT-BioFluo (500 ms exposure time) and the IVIS (1 s exposure time). Optical images obtained with U-SPECT-BioFluo and with IVIS are very similar. For SPECT reconstruction from list mode data, a 20% wide energy window has been set around the 140 keV ^99m^Tc photopeak. The background windows for triple window scatter correction [31] were set to 124 to 136 keV and 166 to 184 keV. POSEM with 16 subsets and 6 iterations was performed for reconstructing 3D SPECT images. The SPECT images were subsequently blurred with a 3D Gaussian low pass filter (FWHM 0.47 mm).

By adjusting the color scale threshold of the fluorescence image, sharp identification of the most superficial part of the capillary can be exchanged for a slight increase in the in-depth visibility. For the low threshold image, the threshold has been set at about the background level of the phantom which is due to e.g. excitation light leakage through the emission filter, fluorescence emission light scatter, and autofluorescence of the phantom [32].

**Figure 5:**
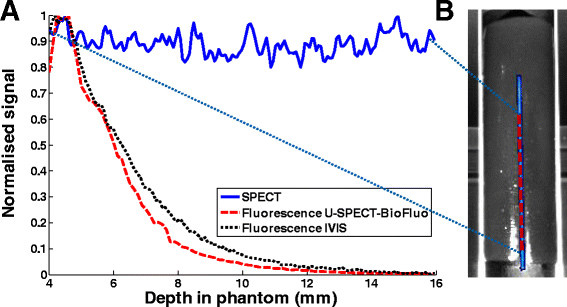
**Effects of depth-dependent attenuation. (A)** Fluorescence signal (phantom background level subtracted) and SPECT signal of capillary versus depth in gel phantom. The maximum SPECT and fluorescence signals have been normalized to 1. **(B)** The positions of the axial line profiles are indicated in the top-view image of the phantom.

In Figure 4A, the SPECT signal is also shown. In the top-view image, the 3D reconstructed SPECT data are vertically summed for 1:1 comparison with the fluorescence measurement. In the side view, the SPECT data are summed horizontally. In Figure 5A, the fluorescence and SPECT signal strength as a function of the capillary depth are shown. Line profiles (~1 mm wide) along the axial direction of the capillary were made from the top-view images for both modalities (Figure 5B). The axial position along these line profiles was converted into the capillary depth by using the inclination angle of the capillary determined from the SPECT side-view image Figure 4A. Both Figures 4A and 5 clearly demonstrate that while the SPECT signal barely suffers from attenuation in this phantom, the fluorescence signal of the uniformly distributed dye (CyAL-5.5_b_) is decreasing with depth. The IVIS and our OI module show a similar attenuation behavior; at 7 mm the signal has already dropped by ~30%, and at 14 mm it is close to the fluorescence background level of the phantom. This decrease can be attributed to the light attenuation and scatter by hemoglobin and water in the phantom. A similar attenuation is observed in tissue [33].

**Figure 6:**
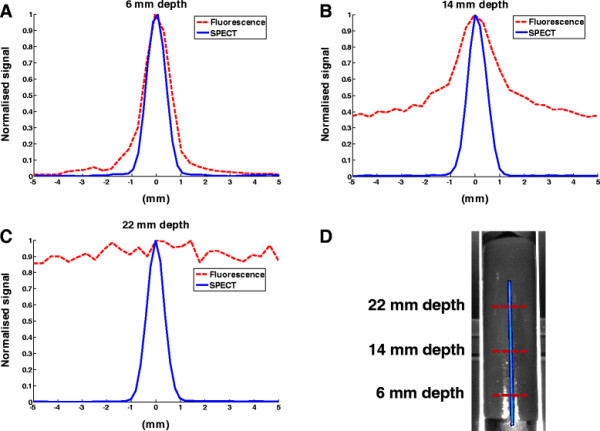
**Comparison of image blurring for SPECT and fluorescence. (A,B,C)** SPECT and fluorescence line profiles orthogonal to the capillary at different depths in the phantom. The maximum signal of each profile has been normalized to 1. **(D)** The positions of the line profiles are indicated in the top-view image.

The resolution curves of the fluorescence and SPECT signals (Figure 6A,B,C) were obtained by making ~1-mm wide line profiles in the top-view images orthogonal to the capillary corresponding to different depths (Figure 6D). Again, the depths have been obtained from the capillary inclination angle. For comparison, these curves have been normalized to their maximum signal. Like the signal strength, the resolution of the fluorescence images also shows severe depth dependence. At 6 mm depth, the width of the fluorescence profile was still close to that of the SPECT profile, namely ~1.2 mm (FWHM). At 14 mm depth, the resolution decreased to ~1.7 mm (FWHM) and the peak value was just 2.5 times the background. Both widths are largely determined by the 0.9 mm diameter of the capillary itself. Around 22 mm depth, the fluorescence signal can no longer be distinguished from the background.

### 3.2 *In vivo* measurements

**Figure 7:**
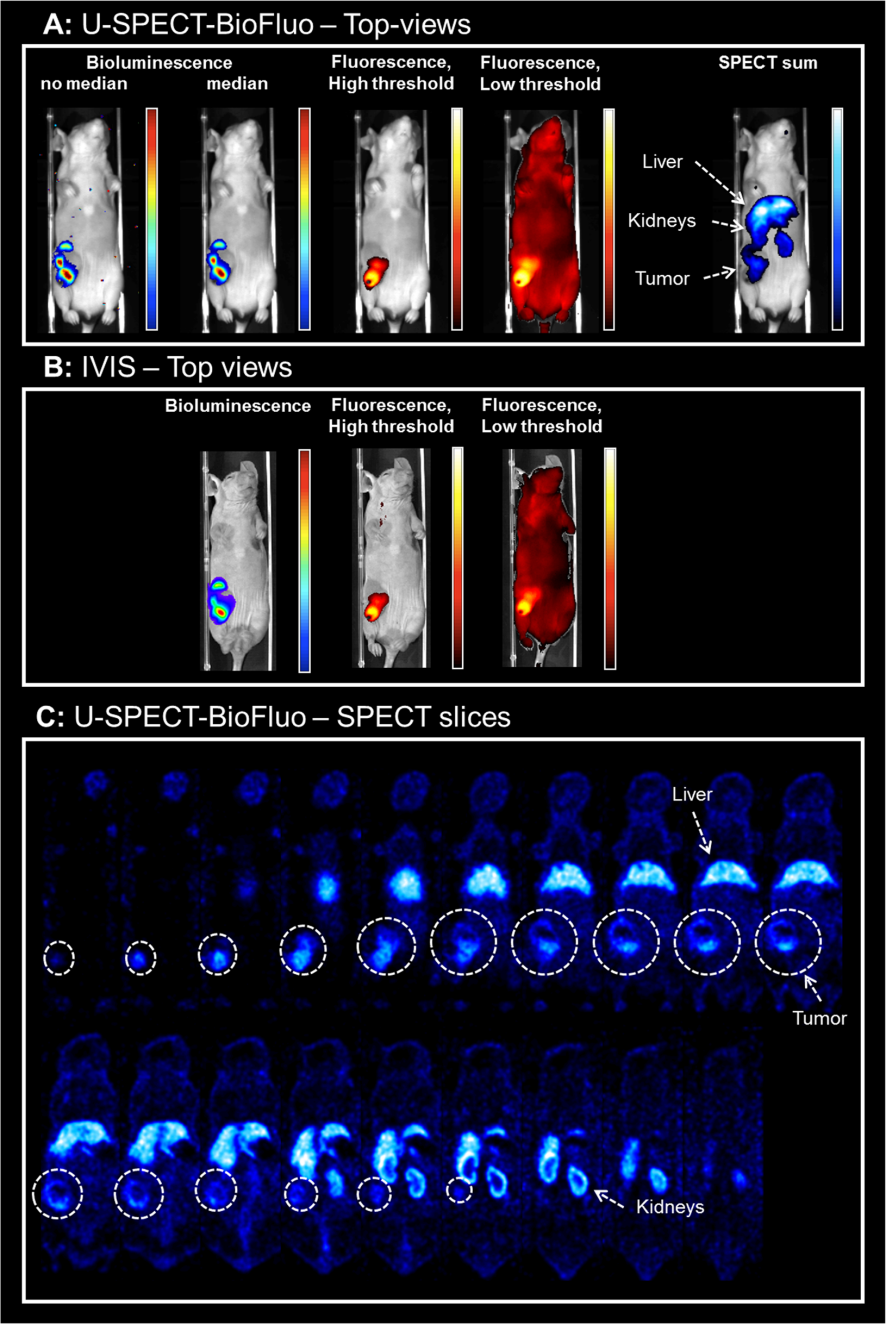
**Imaging results for a mouse with a 4 T1-luc + tumor.** Bioluminescence images are obtained by D-Luciferin injection. Fluorescence and SPECT images are obtained by injection of multimodal tracer ^111^In-RGD-MSAP. **(A)** U-SPECT-BioFluo. Bioluminescent images both with and without median filter applied. **(B)** IVIS. **(C)** U-SPECT-BioFluo: 1-mm thick horizontal SPECT slices (anterior to posterior). Organs have been indicated in the SPECT images. *White circles* indicate the tumor location in the slices.

Figure 7 presents the imaging results obtained from the different modalities in both U-SPECT-BioFluo and IVIS. Figure 7A shows respectively the bioluminescence, fluorescence, and SPECT sum images recorded with U-SPECT-BioFluo. The exposure time for the bioluminescence measurement was 60 s and for the fluorescence measurement 300 ms, as the fluorescent signal was much stronger. On the IVIS (Figure 7B), the exposure times were respectively 30 s and 1 s. The IVIS images are similar to those of the in-house developed OI. The bioluminescence images clearly show the viable tumor component, where luciferase converts D-Luciferin in bioluminescent light.

In the U-SPECT-BioFluo bioluminescence images in Figure 7A, one can notice some hot pixels that may be caused by absorption of ^111^In-based gamma radiation in the CCD chip. This radiation was emitted by the radioactive component of the multimodal tracer ^111^In-RGD-MSAP. A median filter of 4 × 4 pixels has been applied to remove these hot pixels. For the POSEM reconstruction (16 subsets and 6 iterations) of the SPECT scan, a 20% wide energy window has been set around the 171 keV ^111^In photopeak. Background windows of 151 to 167 keV and 205 to 226 keV were chosen for the scatter correction. A 3D Gaussian filter (FWHM 1.18 mm) has been applied after reconstruction.

Looking at the figures, it becomes clear that the signal-depth related effects severely limit the diagnostic utility of the optical images. Nevertheless, the superficially located mammary tumors could be depicted using both optical technologies. Other than in the phantom setup, here the effect of fluorescence threshold changes was minimal. However, in the image representing the summed SPECT slices, the threshold was set at such a level that a tiny part of tumor seems to be ‘missing’ with respect to the optical images. We believe that the intensity of the signal of deeper located cells in SPECT can cause slightly different locations of the tumor compared to the tumor as is visualized by optical imaging: Optical imaging mainly displays the surface of the tracer distribution, while the SPECT shows deeper structures in the mouse very clear as well which affects also the sum image. On the other hand, the SPECT images revealed a substantial uptake of ^111^In-RGD-MSAP in other regions of the mouse, e.g. the liver and kidneys, a finding that correlates with previously reported biodistribution studies [30]. The difference in detectability between the different modalities also becomes apparent when one zooms in on the tumor itself: SPECT images in Figure 7C show heterogeneity of tracer uptake (e.g. an area with less accumulated tracer) that can not be seen in fluorescence images. Earlier studies confirm that microSPECT enables to visualize and quantify tracer heterogeneity in tumors [24],[34].

## 4 Discussion

The rise of optical techniques for molecular imaging purposes has resulted in a demand for dedicated modalities. One of today’s challenges lies in placing optical findings in perspective to nuclear medicine technologies. There is a trend to combine nuclear medicine with supplementary read-outs provided by other modalities. One very practical solution is to integrate multiple modalities on a single platform to prevent deformation issues. For this reason, we developed an optical module that can be provided as an add-on to the U-SPECT-II modality for preclinical nuclear medicine. Using luciferase-gene-modified tumor cells and multimodal tracers that combine radionuclear and optical properties [10]-[12], we incorporated bioluminescence and fluorescence imaging in a 3D SPECT imaging setting.

This initial pilot study underlined that the prototype design (see Figure 1) provided such a small CCD image background that both bioluminescence and fluorescence imaging could be successfully achieved. We used commercially available 40 nm wide bandpass filters specifically tailored to the excitation and emission spectra of the CyAL-5.5_b_ dye with wavelengths only slightly different from the ones used on the IVIS. This small difference may explain the difference in the fluorescence attenuation profiles shown in Figure 5: Since the absorption coefficient for hemoglobin is higher at 624 nm than at 640 nm, the excitation light is somewhat more attenuated in the phantom on our setup than on the IVIS. For future studies with different dyes other commercial filters can be incorporated. The current halogen excitation lamp and CCD camera of the prototype already allow for excitation across the 450- to 800-nm spectrum.

Reasoning that accurate evaluation of the pharmacokinetics and distribution of a new (optical) imaging tracer is of paramount importance for any form of future clinical translation, we have used the in-depth information provided by SPECT to place the optical findings in perspective. In the present study we illustrate that, using a phantom setup and a multimodal angiogenesis tracer (^111^In-RGD-MSAP) in an orthotopic mouse mammary tumor model, the SPECT/bioluminescence/fluorescence combination proposed in this paper may help prevent misconceptions of the tracer’s specificity that could otherwise have resulted from using an optical evaluation only. The genetically engineered bioluminescent signal was confined to the tumor spread, while the fluorescent and radioactive emissions were used to define the distribution of the multimodal tracer used in this study. While the SPECT images show the complete distribution profile of the tracer used [30], the fluorescence images of both our in-house developed OI module as well as the IVIS images, only detected superficial emissions originating from the tumor. SPECT images clearly show tracer uptake e.g. in the liver (see Figure 7). In this case, possible toxicity issues due to unwanted uptake and retention in organs would have been missed with fluorescence imaging.

Where the preclinical SPECT findings are constant with depth, both the optical detectability and resolution degrade as functions of depth (see Figures 5 and 6). Factors contributing to the degradation of optical signals are signal attenuation and scattering - both increase with depth and occur for both the excitation light and emitted signal. In the case of fluorescence imaging, ‘leakage’ of excitation light through the filters and autofluorescence (depends on the tissue type) may raise the background signal. Even with NIR fluorescent dyes such as indocyanine green (ICG), these effects have shown to limit the use of fluorescence to superficial applications [32]. This said, the added value of optical imaging in superficial surgical guidance and/or pathological imaging situations is obvious and is driving the development of optical imaging tracers or multimodal tracers that contain an optical component [11],[12].

In a future version of the OI module, the filter placement could be automated to enable fast switching between different fluorescent emissions, e.g. for multispectral imaging. This could then be combined directly with multi-isotope capabilities of SPECT, to allow for simultaneous evaluation of even multiple multimodal tracers. With UV transparent and more sensitive optics (e.g. larger aperture or deeper CCD cooling), it may become possible to detect much weaker signals such as the Cerenkov luminescence emitted by PET tracers [32]. As the U-SPECT-II can be adapted to perform collimated imaging of PET tracers (VECTor, [35],[36]), this might even enable combined Cerenkov and PET imaging studies, which may help in the interpretation of Cerenkov findings. In addition, the used SPECT can be equipped with CT, further expanding the integration of imaging to a triple or quadruple imaging platform [37].

## 5 Conclusion

We evaluated a prototype optical add-on module that integrates optical imaging with a dedicated small animal SPECT system. This combination allows for complementary and registered imaging of bioluminescent, fluorescent, and SPECT signatures in a single scan session. The high-resolution and highly quantitative (3D) SPECT information about tracer kinetics and/or biodistribution can improve the interpretation of the fluorescence and bioluminescence images.

## Competing interests

The authors declare that they have no competing interests.

## Authors’ contributions

FJB had the initial idea for this multimodal imaging platform, made the initial design of the optical module, participated in the study design, and revised the manuscript. WAM co-designed the optical module for bioluminescence imaging, carried out initial experiments, and revised the manuscript. MNvO and RK integrated the optical module with the U-SPECT-II scanner, added fluorescence imaging, acquired the images of the phantom and *in vivo* study, analyzed the results, and wrote initial versions of the manuscript. FWBvL, TB, and AB designed the phantom and *in vivo* study and revised the manuscript. TB and AB carried out the phantom and *in vivo* experiments. LJ prepared the dye for the *in vivo* study and revised the manuscript. All authors read and approved the final manuscript.

## Authors’ information

FJB is a founder, board member, and shareholder of MILabs B.V.

## References

[B1] Weissleder R Mahmood U Molecular imaging Radiology 2001 219 316 333 10.1148/radiology.219.2.r01ma19316 11323453

[B2] Ramaswamy AK Hamilton M Joshi RV Kline BP Li R Wang P Goergen CJ Molecular imaging of experimental abdominal aortic aneurysms Scientific World Journal 2013 2013 973150 10.1155/2013/973150 23737735 PMC3655677

[B3] Culver J Akers W Achilefu S Multimodality molecular imaging with combined optical and SPECT/PET modalities J Nucl Med 2008 49 169 172 10.2967/jnumed.107.043331 18199608

[B4] Deroose CM De A Loening AM Chow PL Ray P Chatziioannou AF Gambhir SS Multimodality imaging of tumor xenografts and metastases in mice with combined small-animal PET, small-animal CT, and bioluminescence imaging J Nucl Med 2007 48 295 303 17268028 PMC3263830

[B5] Park JM Gambhir SS Multimodality radionuclide, fluorescence, and bioluminescence small-animal imaging Proc IEEE 2005 93 771 783 10.1109/JPROC.2005.844263

[B6] Ivashchenko O Van der Have F Villena J Groen H Ramakers RM Weinans HH Beekman FJ Quarter-mm resolution molecular mouse imaging with U-SPECT+ Mol Imaging 2014 ᅟ ᅟ In Press 10.2310/7290.2014.00053 25429783

[B7] Kuil J Buckle T Oldenburg J Yuan HS Borowsky AD Josephson L van Leeuwen FWB Hybrid peptide dendrimers for imaging of chemokine receptor 4 (CXCR4) expression Mol Pharm 2011 8 2444 2453 10.1021/mp200401p 22085282 PMC3711081

[B8] Luker KE Gupta M Luker GD Bioluminescent CXCL12 fusion protein for cellular studies of CXCR4 and CXCR7 Biotechniques 2009 47 625 632 10.2144/000113126 19594447 PMC4418468

[B9] Van Leeuwen FWB de Jong M Lahoutte T Evangelista L Barbet J Del Vecchio S Schibli R Molecular imaging: the emerging role of optical imaging in nuclear medicine Eur J Nucl Med Mol Imaging 2014 41 2150 2153 10.1007/s00259-014-2845-0 25037872

[B10] Kuil J Velders AH van Leeuwen FWB Multimodal tumor-targeting peptides functionalized with both a radio- and a fluorescent label Bioconjug Chem 2010 21 1709 1719 10.1021/bc100276j 20812730

[B11] Buckle T Chin PTK van Leeuwen FWB (Non-targeted) radioactive/fluorescent nanoparticles and their potential in combined pre- and intraoperative imaging during sentinel lymph node resection Nanotechnology 2010 21 482001 10.1088/0957-4484/21/48/482001 21063057

[B12] Azhdarinia A Ghosh P Ghosh S Wilganowski N Sevick-Muraca EM Dual-labeling strategies for nuclear and fluorescence molecular imaging: a review and analysis Mol Imaging Biol 2012 14 261 276 10.1007/s11307-011-0528-9 22160875 PMC3346941

[B13] van den Berg NS Valdes-Olmos RA van der Poel HG van Leeuwen FWB Sentinel lymph node biopsy for prostate cancer: a hybrid approach J Nucl Med 2013 54 493 496 10.2967/jnumed.112.113746 23492883

[B14] Brouwer OR Buckle T Vermeeren L Klop WMC Balm AJM van der Poel HG van Rhijn BW Horenblas S Nieweg OE van Leeuwen FWB Olmos RAV Comparing the hybrid fluorescent-radioactive tracer indocyanine green-Tc-99 m-nanocolloid with Tc-99 m-nanocolloid for sentinel node identification: a validation study using lymphoscintigraphy and SPECT/CT J Nucl Med 2012 53 1034 1040 10.2967/jnumed.112.103127 22645297

[B15] Cao L Peter J Investigating line- versus point-laser excitation for three-dimensional fluorescence imaging and tomography employing a trimodal imaging system J Biomed Opt 2013 18 066015 10.1117/1.JBO.18.6.066015 23797896

[B16] Li CQ Yang YF Mitchell GS Cherry SR Simultaneous PET and multispectral 3-dimensional fluorescence optical tomography imaging system J Nucl Med 2011 52 1268 1275 10.2967/jnumed.110.082859 21810591 PMC4557773

[B17] Tan IC Lu YJ Darne C Rasmussen JC Zhu BH Azhdarinia A Yan SK Smith AM Sevick-Muraca EM Fluorescence-enhanced optical tomography and nuclear imaging system for small animals Multimodal Biomed Imag VII, Proc SPIE 2012 8216 1

[B18] Lu Y Yang K Zhou K Pang B Wang G Ding Y Zhang Q Han H Tian J Li C Ren Q An integrated quad-modality molecular imaging system for small animals J Nucl Med 2014 55 1375 1379 10.2967/jnumed.113.134890 24947062

[B19] Solomon M Nothdruft RE Akers W Edwards WB Liang KX Xu BG Suddlow GP Deghani H Tai YC Eggebrecht AT Achilefu S Culver JP Multimodal fluorescence-mediated tomography and SPECT/CT for small-animal imaging J Nucl Med 2013 54 639 646 10.2967/jnumed.112.105742 23447655 PMC3956308

[B20] Darne C Lu YJ Sevick-Muraca EM Small animal fluorescence and bioluminescence tomography: a review of approaches, algorithms and technology update Phys Med Biol 2014 59 R1 R64 10.1088/0031-9155/59/1/R1 24334634

[B21] van der Have F Vastenhouw B Ramakers RM Branderhorst W Krah JO Ji CG Staelens SG Beekman FJ U-SPECT-II: an ultra-high-resolution device for molecular small-animal imaging J Nucl Med 2009 50 599 605 10.2967/jnumed.108.056606 19289425

[B22] Branderhorst W Vastenhouw B van der Have F Blezer ELA Bleeker WK Beekman FJ Targeted multi-pinhole SPECT Eur J Nucl Med Mol Imaging 2011 38 552 561 10.1007/s00259-010-1637-4 21063706 PMC3034876

[B23] Befera NT Badea CT Johnson GA Comparison of 4D-MicroSPECT and MicroCT for murine cardiac function Mol Imaging Biol 2014 16 235 245 10.1007/s11307-013-0686-z 24037175 PMC4061569

[B24] Zhou Y Shao GQ Liu S Monitoring breast tumor lung metastasis by U-SPECT-II/CT with an integrin alpha(v)beta(3)-targeted radiotracer Tc-99 m-3P-RGD(2) Theranostics 2012 2 577 588 10.7150/thno.4443 22737193 PMC3381346

[B25] Branderhorst W Vastenhouw B Beekman FJ Pixel-based subsets for rapid multi-pinhole SPECT reconstruction Phys Med Biol 2010 55 2023 2034 10.1088/0031-9155/55/7/015 20299722

[B26] van der Have F Vastenhouw B Rentmeester M Beekman FJ System calibration and statistical image reconstruction for ultra-high resolution stationary pinhole SPECT IEEE Trans Med Imaging 2008 27 960 971 10.1109/TMI.2008.924644 18599401

[B27] Shao FW Yuan HS Josephson L Weissleder R Hilderbrand SA Facile synthesis of monofunctional pentamethine carbocyanine fluorophores Dyes Pigm 2011 90 119 122 10.1016/j.dyepig.2010.12.008 21475610 PMC3070263

[B28] Pleijhuis RG Langhout GC Helfrich W Themelis G Sarantopoulos A Crane LMA Harlaar NJ de Jong JS Ntziachristos V van Dam GM Near-infrared fluorescence (NIRF) imaging in breast-conserving surgery: assessing intraoperative techniques in tissue-simulating breast phantoms Eur J Surg Oncol 2011 37 32 39 10.1016/j.ejso.2010.10.006 21106329

[B29] Buckle T Chin PTK van den Berg NS Loo CE Koops W Gilhuijs KGA van Leeuwen FWB Tumor bracketing and safety margin estimation using multimodal marker seeds: a proof of concept J Biomed Opt 2010 15 056021 10.1117/1.3503955 21054115

[B30] Bunschoten A Buckle T Visser NL Kuil J Yuan HS Josephson L Vahrmeijer AL van Leeuwen FWB Multimodal interventional molecular imaging of tumor margins and distant metastases by targeting alpha v beta 3 Integrin Chembiochem 2012 13 1039 1045 10.1002/cbic.201200034 22505018 PMC3498954

[B31] Ogawa K Harata Y Ichihara T Kubo A Hashimoto S A practical method for position-dependent compton-scatter correction in single photon-emission Ct IEEE Trans Med Imaging 1991 10 408 412 10.1109/42.97591 18222843

[B32] Chin PTK Welling MM Meskers SCJ Olmos RAV Tanke H van Leeuwen FWB Optical imaging as an expansion of nuclear medicine: cerenkov-based luminescence vs fluorescence-based luminescence Eur J Nucl Med Mol Imaging 2013 40 1283 1291 10.1007/s00259-013-2408-9 23674205

[B33] Chin PT Beekman CA Buckle T Josephson L van Leeuwen FW Multispectral visualization of surgical safety-margins using fluorescent marker seeds Am J Nucl Med Mol Imaging 2012 2 151 162 23133810 PMC3477729

[B34] Branderhorst W Blezer EL Houtkamp M Ramakers RM van den Brakel JH Witteveen H van der Have F van Andel HAG Vastenhouw B Wu C Stigter-van Walsum M van Dongen GAMS Viergever MA Bleeker WK Beekman FJ Three-dimensional histologic validation of high-resolution SPECT of antibody distributions within xenografts J Nucl Med 2014 55 830 837 10.2967/jnumed.113.125401 24686779

[B35] Goorden MC van der Have F Kreuger R Ramakers RM Vastenhouw B Burbach JPH Booij J Molthoff CFM Beekman FJ VECTor: a preclinical imaging system for simultaneous submillimeter SPECT and PET J Nucl Med 2013 54 306 312 10.2967/jnumed.112.109538 23077113

[B36] Walker MD Goorden MC Dinelle K Ramakers RM Blinder S Shirmohammad M van der Have F Beekman FJ Sossi V Performance assessment of a preclinical PET scanner with pinhole collimation by comparison to a coincidence-based small-animal PET scanner J Nucl Med 2014 55 1368 1374 10.2967/jnumed.113.136663 24904110

[B37] Vaissier PEB Wu C Beekman FJ Integration of small animal SPECT and PET with other imaging modalities Tijdschrift voor Nucleaire Geneeskunde 2012 35 961 967

